# Robust effector memory features of human T-bet^hi^ B cells induced by repeated mRNA vaccination

**DOI:** 10.1016/j.isci.2026.114804

**Published:** 2026-01-27

**Authors:** Jeongsoo Lee, Seunghwan Son, Youseung Chung, Sung-Dong Cho, Ji-Soo Kwon, Seongman Bae, Joon Seok, Baekgyu Choi, Inkyung Jung, Ji Eun Oh, Eui-Cheol Shin, Sung-Han Kim, Su-Hyung Park

**Affiliations:** 1 Graduate School of Medical Science and Engineering, Korea Advanced Institute of Science and Technology (KAIST), Daejeon, Republic of Korea; 2 Department of Infectious Diseases, Asan Medical Center, University of Ulsan College of Medicine, Seoul, Republic of Korea; 3 Department of Dermatology, College of Medicine, Chung-Ang University, Seoul, Republic of Korea; 4 Department of Life Science, Korea Advanced Institute of Science and Technology (KAIST), Daejeon, Republic of Korea

**Keywords:** Immunology, Immune response, Virology

## Abstract

The heterogeneity of memory B cells in response to repetitive cognate antigen challenges remains to be fully elucidated. Here, we identified a transcriptionally distinct cluster of T-bet^hi^ B cells, among SARS-CoV-2 RBD-specific B cells in PBMCs from healthy individuals vaccinated with the BNT162b2 SARS-CoV-2 mRNA vaccine. Our findings indicate that T-bet^hi^ B cells can be defined by a combination of CD11c and FcRL5 receptors, and are distinguished by distinct gene regulatory networks associated with effector functions. Notably, these T-bet^hi^ B cells were affinity-matured and exhibited rapid differentiation into antibody-secreting cells (ASCs) producing neutralizing antibodies comparable to classical memory B cells, underscoring their role in early recall responses. Taken together, these findings illuminate the potent effector memory roles of T-bet^hi^ B cells in adaptive immunity following vaccinations.

## Highlights

• Repeated vaccination induces affinity-matured T-bet^hi^ B cells with effector traits
• Vaccine-induced T-bet^hi^ B cells are defined by CD11c, FcRL5, and a T-bet gene network
• T-bet^hi^ B cells rapidly form neutralizing antibody-secreting cells upon recall

## Graphical abstract

**Figure:**
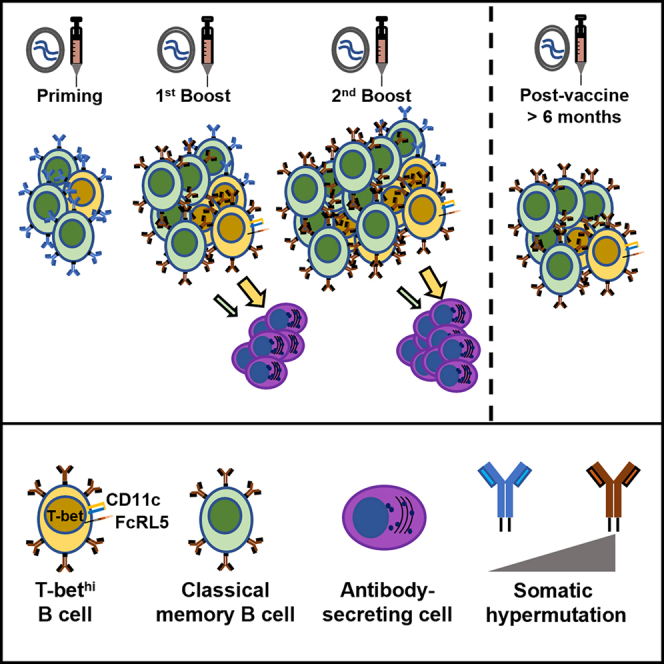

## Introduction

Memory B cells are a central component of humoral immunity, providing rapid recall responses against previously encountered antigens.^1^ Previous studies have revealed that memory B cells are heterogeneous, comprising subsets with distinct phenotypes and effector functions.^2^^,^^3^ This complexity challenges the classical paradigm of CD27^+^ class-switched memory B cells, as unconventional subsets lacking CD27^4^^,^^5^ or exhibiting atypical surface markers have been identified in human blood and lymphoid tissues.^6^

One such subset is commonly defined by high expression of the transcription factor T-bet (TBX21). These T-bet^hi^ B cells, also termed as atypical B cells, age-associated B cells (ABCs), or double-negative 2 (DN2) cells, expand during chronic infections and autoimmune diseases.^7^^,^^8^^,^^9^^,^^10^ These populations have been described under different names and defined by combinations of surface receptors such as CD21^−^CD27^−^, or by expression of CD11c and/or FcRL5. T-bet expression represents an important, though not exclusive, characteristic linked to their functional and differentiation programs. Moreover, T-bet^hi^ B cells have been observed within both CD27^−^ and CD27^+^ memory compartments.^4^^,^^5^ Despite their heterogeneous features, multiple studies have demonstrated that the differentiation of this subset is driven by inflammatory signals, such as IFN-γ, BCR engagement, and Toll-like receptors (TLR) ligands,^11^^,^^12^^,^^13^ highlighting the need for more detailed dissection and classification. Accumulating evidence further indicates that T-bet^hi^ B cells, initially described as dysfunctional or “exhausted” due to impaired proliferation and BCR responsiveness, can also contribute to effector responses under certain contexts.^8^^,^^14^^,^^15^

Recent studies have identified ZEB2 as a transcriptional driver of this lineage. ZEB2 represses germinal center programs while promoting the atypical cell fate, thereby sustaining germinal center output during persistent antigen exposure. In both mouse and human systems, ZEB2 expression is essential for CD11c^+^ atypical B cell differentiation and function, establishing it as a central regulator of the T-bet^hi^ program,^10^^,^^16^ suggesting rather complex and unique nature of this subset.

In autoimmune diseases such as systemic lupus erythematosus, T-bet^hi^ B cells act as precursors of antibody-secreting cells (ASCs) and promote autoantibody production.^17^ In severe viral infections, including COVID-19, they arise via extrafollicular activation and are associated with both neutralizing antibody responses and immunopathology.^18^^,^^19^ In contrast, vaccination studies have suggested that CD11c^+^ T-bet^hi^ B cells participate in early recall responses, displaying transcriptional and metabolic programs predictive of durable humoral immunity,^20^^,^^21^ suggesting that this subset can exert robust effector memory functions depending on the context. Furthermore, a recent mouse study showed that T-bet is required for the maintenance and functional programming of virus-induced memory B cells in lymph nodes and lungs.^22^ Consistent with these findings, functional studies have demonstrated that T-bet^hi^ B cells integrate strong inhibitory receptor signaling; for instance, high expression of FcγRIIB selectively limits their responsiveness to soluble antigens while preserving robust activation against membrane-associated antigens, underscoring their specialized effector potential.^23^

Despite these important insights, the molecular and functional features of T-bet^hi^ memory B cells under controlled conditions of repeated vaccination remain incompletely defined. In particular, it is unclear whether these cells undergo robust affinity maturation following repeated antigen exposure and how they compare to classical memory subsets in vaccine-induced recall responses. Furthermore, their heterogeneity at the single-cell level in the setting of repeated vaccination has not been systematically characterized.

Here, we analyzed SARS-CoV-2 receptor-binding domain (RBD)-specific memory B cells from individuals who received multiple doses of the BNT162b2 SARS-CoV-2 mRNA vaccine. Using multi-omics single-cell sequencing, gene regulatory network analysis, immunophenotyping, and functional assays, we characterize the effector memory features of T-bet^hi^ memory B cells. Our findings reveal their unique gene regulatory networks, robust affinity maturation, rapid differentiation potential, and potential contribution to vaccine-induced recall immunity.

## Results

### Multiple COVID-19 mRNA vaccinations induce T-bet^hi^ SARS-CoV-2 RBD-specific B cells

**Figure 1:**
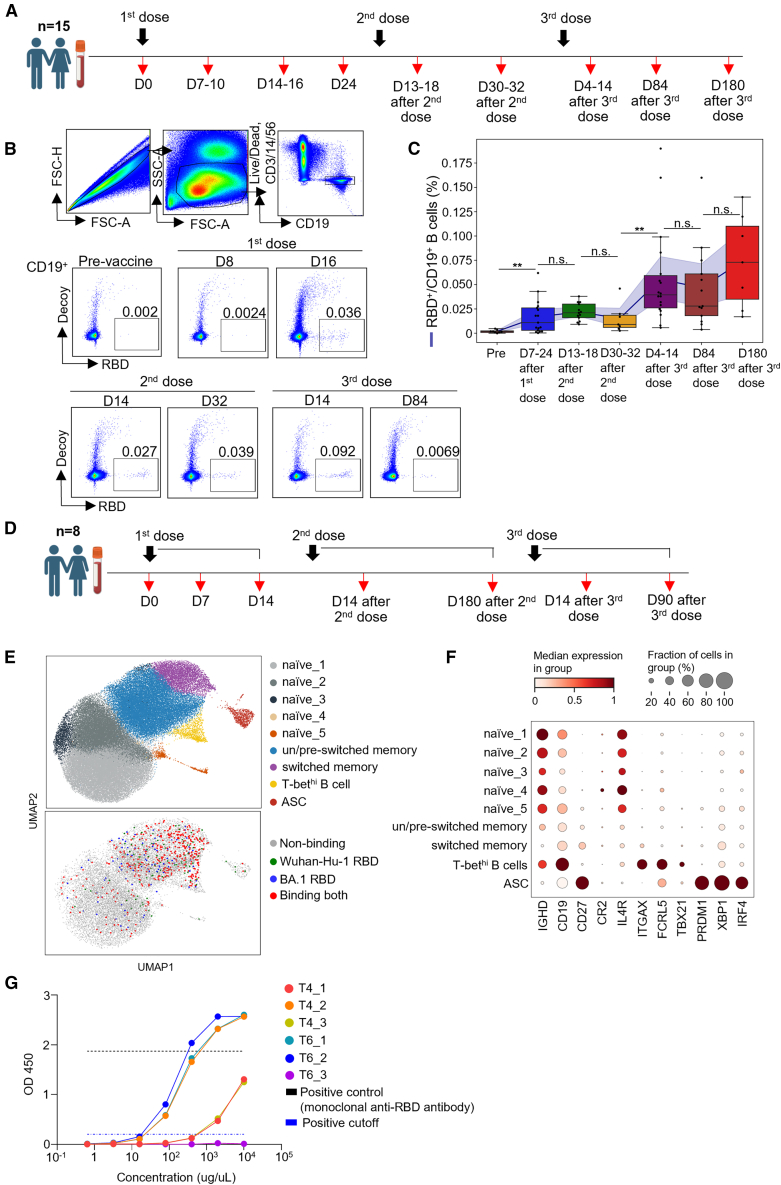
Repeated COVID-19 mRNA vaccinations induce T-bet^hi^ SARS-CoV-2 RBD-specific B cells (A) Timeline of mRNA vaccinations and blood sample acquisition from healthy medical personnel. (B) Representative plot of gating strategy capturing RBD-specific B cells from peripheral blood mononuclear cells (PBMCs). (C) Kinetics of the frequency of RBD-specific B cells across multiple mRNA vaccinations. (D) Timeline of mRNA vaccinations and the acquisition of blood samples used for LIBRA-seq analysis. (E) Uniform manifold approximation and projection (UMAP) showing multiple transcriptionally distinct clusters identified by LIBRA-seq (top), and an overlay of RBD-specific cells on UMAP clusters (bottom). (F) Representative marker genes used to annotate each cluster. (G) Indirect ELISA assay with recombinant antibodies made from VDJ sequences of D14 T-bet^hi^ B cells after 2^nd^ and 3^rd^ vaccination. Mann-Whitney U test, ∗*p* < 0.05, ∗∗*p* < 0.01, ∗∗∗*p* < 0.001, ∗∗∗∗*p* < 0.0001; n.s., non-significant. Pooled data are represented as median ±SD.

To investigate the dynamics of memory B cells in response to multiple cognate antigen challenges, we recruited a cohort of healthy medical personnel who had not contracted COVID-19 at the time of recruitment, and collected their PBMCs over the course of three Pfizer BNT162b2 mRNA vaccinations (Figure 1A). To isolate SARS-CoV-2 RBD-specific B cells from PBMCs, we produced Wuhan-Hu-1 SARS-CoV-2 RBD-bound fluorochrome-conjugated streptavidin, as previously described.^24^ Across the three vaccinations, RBD-specific B cells were successfully isolated from vaccine recipients who had not contracted symptomatic COVID-19 up to the last blood collection time point, and these cells showed increasing frequency and persistent maintenance up to six months after the third dose (Figures 1B and 1C). To further characterize memory B cells induced by BNT162b2, in an unbiased fashion, we employed LIBRA-seq^25^ with single-cell B cell receptor sequencing (BCR-seq), using a mixture of two tetramers comprising oligonucleotide-conjugated streptavidin bound with Wuhan-Hu-1 RBD and Omicron (BA.1) RBD, respectively. From the volunteer cohort, we selected peripheral blood mononuclear cells collected at seven time points from eight individuals who had not experienced a breakthrough infection up to the last time point (Figure 1D). CD19^+^-sorted and tetramer^+^ cell-enriched B cells were used for sequencing. After quality control, a total of 76,255 B cells were acquired, with 598 RBD-binding cells, including cells from all eight subjects (Figures S1A and S1B), mainly distributed over memory clusters (Figure 1E). Each cluster was annotated using representative marker genes, and we identified a distinct cluster in proximity to memory cell clusters (Figure 1E, yellow), which we annotated as “T-bet^hi^ B cell” because it was characterized by expressions of CD19, ITGAX, FCRL5, and TBX21 (Figure 1F). In addition, to verify that the RBD-tetramer-binding B cells are indeed RBD-specific, we generated recombinant IgG antibodies using the VDJ sequences of RBD-binding T-bet^hi^ B cells isolated at day 14 after both the 2^nd^ and 3^rd^ vaccinations. A total of six sequences—three from day 14 after the second vaccination and three from day 14 after the third vaccination—were selected from the top three frequency bins of the LIBRA-seq score distribution for the Wuhan-Hu-1 tetramer to ensure representativeness (Figure S1C, arrows). Indirect ELISA performed with a pre-made kit (GenScript, New Jersey, USA) revealed that 5 out of 6 antibodies exhibited significant binding to plate-coated SARS-CoV-2 RBD (Figure 1G), thereby validating that the tetramer-binding cells were RBD-specific B cells.

Overall, these findings suggest that multiple COVID-19 mRNA vaccinations induced T-bet^hi^ SARS-CoV-2 RBD-specific memory B cells.

### T-bet^hi^ B cells are transcriptionally distinct with unique gene regulatory networks and heterogeneous subgroups

**Figure 2:**
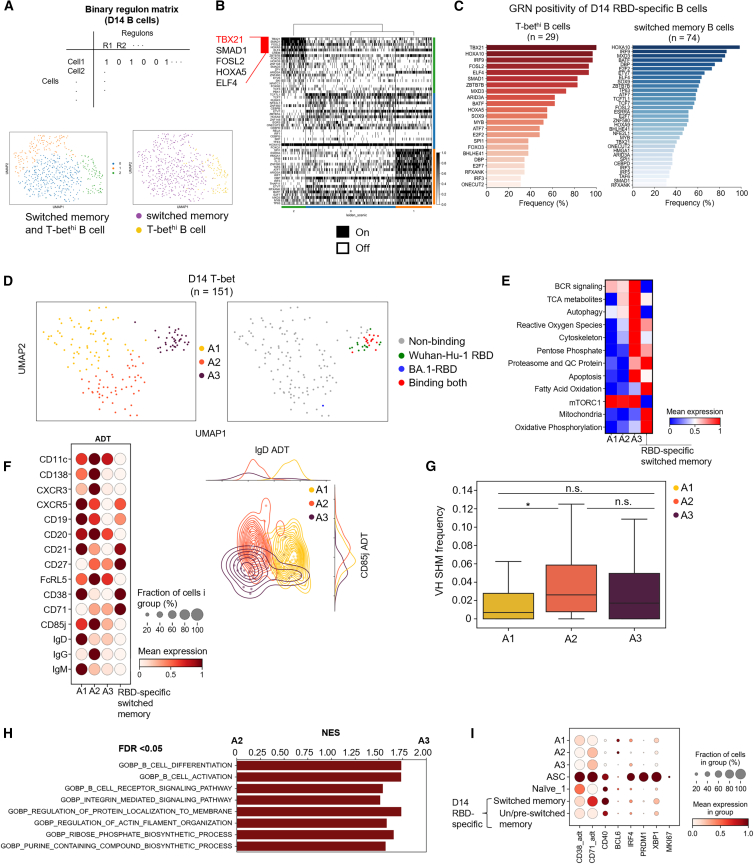
T-bet^hi^ B cells are transcriptionally distinct with unique gene regulatory networks and heterogeneous subgroups (A) Unbiased clustering of the binary regulon matrix generated by SCENIC analysis reveals that T-bet^hi^ B cells are distinct from classical switched memory B cells. (B) Binary heatmap showing cluster-specific gene regulatory networks (GRNs). Red bar indicates the top 5 most-specific GRNs active in T-bet^hi^ B cells. (C) Bar plot showing active GRNs in receptor-binding domain (RBD)-specific T-bet^hi^ and switched memory B cells. GRNs positive in over 30% of cells in the cluster are shown. (D) T-bet^hi^ B cells are further subclustered into three different clusters based on gene expression and antibody-derived tag (ADT) data (except tetramers and Ig ADT). (E) Module scores of metabolic module gene sets in each subcluster. (F) Each subset shows differential expression of surface receptors, and is best delineated by a combination of IgD and CD85j expression. (G) Heavy chain somatic hypermutation frequency among T-bet^hi^ B cells subclusters. (H) Significantly enriched gene sets from GSEA with curated terms from gene ontology: biological process. (I) Standardized expression level of CD38/CD71 (as ADT), IRF4, PRDM1, XBP1, and MKI67 in naive, RBD-specific un/pre-switched memory, switched memory, T-bet^hi^ B cell subclusters and all ASCs from D14 after 2^nd^ or 3^rd^ vaccination. Mann-Whitney U test, ∗*p* < 0.05, ∗∗*p* < 0.01, ∗∗∗*p* < 0.001, ∗∗∗∗*p* < 0.0001; n.s., non-significant. Pooled data are represented as mean ± SD.

It is difficult to uniformly characterize T-bet^hi^ B cells because different characteristics and functions have been reported depending on the disease context. Here, we aimed to elucidate the immunological characteristics of T-bet^hi^ B cells induced by COVID-19 mRNA vaccinations at D14 after each vaccination, which were distinct in the unbiased clustering process of sampled B cells. First, we examined whether T-bet^hi^ B cells were governed by specific gene regulatory networks that distinguish them from switched memory B cells, rather than simply differentially expressed genes. To this end, we performed SCENIC^26^ analysis to detect gene regulatory networks (GRNs), called regulons, among sampled B cells, and their activity in each cell (Table S2). After identifying regulons, the activities of the regulons in each cell were used to construct a binary cell-by-regulon matrix using AUCell analysis (Figure 2A, top). The total switched memory and T-bet^hi^ B cells at D14 after the 2^nd^ and 3^rd^ vaccinations as regulon activity vectors in the binary matrix were clustered onto UMAP space, to categorize cells in terms of GRNs (Figure 2A, bottom-left). This yielded the identification of three clusters. When we overlaid previous annotations, the T-bet^hi^ B cells were clearly distinguished from switched memory B cell clusters (Figure 2A, bottom-right; Figure 2B). Examining the specific activities of GRNs in each cluster revealed that the TBX21 GRN was active in 93.7% of total T-bet^hi^ B cells, compared to only 18.4% of total switched memory B cells (Figure S2A). Among RBD-specific B cells, the TBX21 regulon was active in 100% of 29 T-bet^hi^ B cells, and in 37.8% of 74 switched memory B cells. These findings suggest that the T-bet-associated GRN is indeed a key distinct transcriptional feature of T-bet^hi^ B cells (Figure 2C). The activities of subset-specific GRNs showed overall correlation between 2^nd^ and 3^rd^ vaccination (Figure S2B) and both subsets shared certain GRNs such as ELF4 that regulates cell cycle^27^ and IRF9, a key mediator of type I interferon signaling.^28^ In addition, RBD-specific T-bet^hi^ B cells upregulated ITGAX, FCRL5, GPR138, and CXCR3 compared with RBD-specific switched memory B cells (Figure S2D), suggesting differential homing pattern of this subset. The expression of these genes was correlated significantly with TBX21 expression (Figure S2E).

Next, to investigate the heterogeneity of T-bet^hi^ B cells, we performed weighted nearest neighbor (WNN) analysis to subcluster the T-bet^hi^ B cells cluster using both transcriptome and antibody-derived tags (ADT) data.^29^ When clustering cell, tetramer ADTs and Ig ADTs were excluded to avoid potential bias due to antigen specificity per se. Since previous studies have reported that activated naive B cells also exhibit high T-bet expression and associated gene regulatory networks, which was shown to represent precursors of pathogenic DN2 cells in SLE,^17^^,^^30^ we also sought to delineate T-bet^hi^ B cells from these activated naive B cells. Interestingly, the T-bet^hi^ B cell cluster was further divided into three distinct subclusters, annotated as A1, A2, and A3, with the A3 cluster being almost exclusively enriched with RBD-specific cells (Figure 2D). To further characterize these subclusters’ transcriptomes, we calculated module scores of gene sets that were curated as signature metabolic features of T-bet^hi^ B cells previously detected in flu-vaccinated individuals.^21^ In contrast to the A1 and A2 clusters, cells in the A3 cluster exhibited high scores for most analyzed metabolic features, indicating that the RBD-specific cells in the A3 cluster showed metabolic changes related to cognate antigen exposure. Interestingly, RBD-specific switched memory B cells also exhibited enrichment of certain metabolic feature gene sets, but showed a pattern clearly distinct from the A3 cluster cells (Figure 2E), with these two different cell types having both common and distinct metabolic features. Additionally, each subcluster showed differential surface receptor expressions. A1 cells showed the highest frequency of IgD and IgM isotypes in BCR sequencing (Figure S3A) and ADT (Figure 2F, left) as well, along with higher BACH2 expression (Figure S3D) and lower somatic hypermutation frequency (Figure 2G). These are characteristic features of activated naive B cells, as reported in a previous study.^17^ Therefore, we re-annotated A1 as “activated naive B cells” and A2 and A3 as “T-bet^hi^ B cells”. Notably, A2 and A3 differed in their expression of CD85j (Figure 2F, right), which serves as an inhibitory receptor and immune checkpoint with ITIM motifs at the cytoplasmic tail.^31^ This is consistent with a previous study^4^ that demonstrated heterogeneity of T-bet^hi^ B cells based on CD85j expression. Since high CD85j expression has been reported in most T-bet^hi^ B cells found in chronic HIV infection and malaria patients,^4^^,^^32^ these observations suggest that these CD85j^lo^ A3 cluster cells, which are predominantly RBD-binding and likely generated by SARS-CoV-2 vaccination, may represent a distinct subset from those observed in chronic diseases. We also examined the heavy-chain somatic hypermutation frequency of each subcluster and found comparable mutation frequencies between A2 cluster cells and the RBD-specific cells of the A3 cluster (Figure 2G), indicating that both subclusters are antigen-experienced. Gene set enrichment analysis (GSEA) further revealed that A3 cluster cells were significantly enriched in gene sets related to BCR signaling, activation, and differentiation (Figure 2H), suggesting that some of these cells may be generated or activated by vaccination.

Next, we examined whether the T-bet^hi^ B cells reflect a transient activation state of recently stimulated memory B cells rather than a stable subset. We analyzed the expression of genes and surface receptors associated with acute activation or plasmablast differentiation—IRF4, CD40, BCL6, PRDM1, XBP1, MKI67, and the activation markers CD38 and CD71 (ADT). None of these genes or proteins were significantly upregulated in any T-bet^hi^ subpopulation compared with RBD-specific switched memory B cells (Figure 2I). This absence of acute activation or proliferation signatures indicates that T-bet^hi^ B cells are less likely to represent a population of recently activated memory cells. These data support the interpretation that T-bet^hi^ B cells correspond to a stably differentiated memory phenotype with a potentially distinct activation trajectory.

Taken together, these results indicate that T-bet^hi^ B cells are characterized by a distinct gene regulatory configuration, in which the T-bet-associated network represents the most specific and defining transcriptional program. The heterogeneity observed within the T-bet^hi^ cluster suggests that these cells encompass multiple activation or differentiation trajectories, which may account for the variable functional properties reported for T-bet^hi^ B cells across different immunological contexts.

### T-bet^hi^ B cells expanded by multiple mRNA vaccinations can be defined by a combination of CD11c and FcRL5 receptors

**Figure 3:**
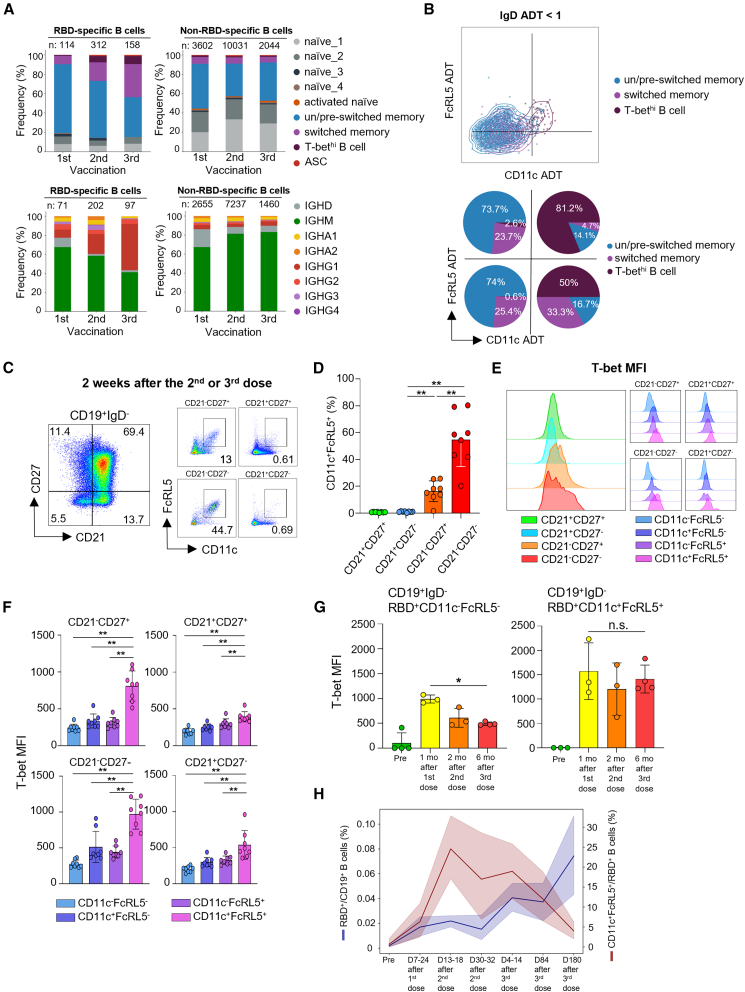
T-bet^hi^ B cells expanded by multiple mRNA vaccinations can be defined by a combination of CD11c and FcRL5 receptors (A) Following multiple vaccinations, the memory compartment significantly expands and switches immunoglobulin class, among RBD-specific B cells but not non-RBD-specific B cells. (B) The T-bet^hi^ B cell cluster is distinguished from classical memory clusters by a combination of CD11c and FcRL5 ADTs. (C) Representative plot of frequencies of B cells expressing both CD11c and FcRL5 among four subsets classified by CD21 and CD27 expressions. (D) Summarized data regarding CD11c^+^FcRL5^+^ B cell frequencies in each subpopulation. (E) Representative plot of T-bet expression, showing higher T-bet mean fluorescence intensity (MFI) in CD21^−^ activated B cell subpopulations. (F) Summarized T-bet MFI data according to CD11c and FcRL5 expression in cells of each subpopulation. (G) Summarized T-bet MFI data of RBD-specific CD11c^+^FcRL5^+^ and CD11c^−^FcRL5^-^ B cells in pre-vaccination, 1 month after 1^st^ vaccination, 2 months after 2^nd^, and 6 months after 3^rd^ vaccination, respectively. (H) Flow cytometry analysis showing kinetics of the frequency of CD11c^+^FcRL5^+^ B cells among RBD^+^ B cells, across multiple vaccinations. Mann-Whitney U test for Figures 3D–3F and Kruskal-Wallis test for Figure 3G, ∗*p* < 0.05, ∗∗*p* < 0.01, ∗∗∗*p* < 0.001, ∗∗∗∗*p* < 0.0001; n.s., non-significant. Pooled data are represented as mean ± SD.

The LIBRA-seq data revealed that the frequency of both switched memory and T-bet^hi^ B cells increased among RBD-specific B cells following each boost vaccination, whereas this trend was not observed among non-RBD-specific B cells (Figure 3A, top). RBD-specific B cells also exhibited isotype switching, mainly toward IgG1 and IgG2, which increased with each boost vaccination among RBD-specific B cells, and was not observed in non-RBD-specific B cells (Figure 3A, bottom).

To confirm whether transcriptionally defined T-bet^hi^ B cells also had distinct surface phenotypes—including those reported in previous studies^33^^,^^34^—we investigated which ADTs best delineated the T-bet^hi^ B cell cluster. To systemically filter ADTs according to their characterizing power, we employed a machine learning model, called SelectKBest^35^ which scored each ADT in terms of its importance in distinguishing the T-bet^hi^ B cell cluster. This scoring revealed that CD11c and FcRL5 were the top two most important features defining the T-bet^hi^ B cell cluster (Figure S4A). Additionally, among IgD ADT-low memory cells (Figure S4B), T-bet^hi^ B cells were distinguished based on higher values of CD11c and FcRL5 ADTs (Figure 3B, top). An *in silico* gating strategy for CD11c and FcRL5 ADT double positivity exhibited the highest positive predictive value for capturing transcriptionally defined T-bet^hi^ B cells (Figure 3B, bottom).

We next examined whether this definition could capture T-bet^hi^ B cells that were induced and recalled in response to multiple mRNA vaccinations *ex vivo*. To this end, we performed multicolor flow cytometry to analyze expressions of the surface receptors CD11c and FcRL5, the transcription factor T-bet, and canonical markers (e.g., CD21 and CD27) in isotype-switched IgD^–^ B cells among D14 PBMCs after the 2^nd^ or 3^rd^ vaccination dose. Common definitions of T-bet^hi^ B cells include CD21^–^CD27^–^ B cells. However, low CD21 expression also serves as an activation marker in B cells^2^ and has been reported to work synergistically with FcRL5 in B cell activation^36^; therefore, this definition may potentially bias the characterization of a certain memory B cell subset. Furthermore, a previous study demonstrated that CD27^+^ B cells also express CD11c and FcRL5 in the TTCF (tetanus toxoid C fragment)-specific B cells of individuals who received a tetanus vaccine.^34^ Notably, the use of only T-bet expression as a defining feature complicates further *ex vivo* or *in vitro* analysis, due to the necessity of intracellular staining that inevitably kills cells. Interestingly, in all four groups classified based on CD21 and CD27, we found B cells expressing CD11c and FcRL5, with varying frequencies, indicating T-bet^hi^ B cells encompass both CD27^+^ and CD27^−^ populations, in line with previous studies^4^^,^^5^ (Figures 3C and 3D). More importantly, CD11c/FcRL5 double positivity best delineated T-bet^hi^ B cells in every group (Figures 3E and 3F). Next, we asked whether this phenotypic pattern is also observed in RBD-specific B cells. To address this, we analyzed the same phenotypes in RBD-tetramer-binding B cells from the same individuals. Consistent with the previous results, CD11c^+^FcRL5^+^ B cells were most frequent within the CD21^−^CD27^−^ subpopulation but were also detected in the other three subpopulations, with particularly substantial representation in the CD21^−^CD27^+^ subset (Figures S5A and S5B). The T-bet MFI values followed the same trend as in total B cells, demonstrating that CD11c/FcRL5 double positivity can serve as a marker of T-bet^hi^ B cells (Figure S5C). This pattern was maintained in RBD-specific B cells at 1, 2, and 6 months after each vaccination, indicating that T-bet^hi^ B cells consistently retain high T-bet expression along with CD11c and FcRL5 expression (Figure 3G). Based on these results, we further gated RBD-specific B cells with CD11c and FcRL5, and detected robust expansion of T-bet^hi^ B cells after each boost vaccination, and a sharp decrease of CD11c^+^FcRL5^+^ cells among RBD-specific B cells (Figure 3H, red line). This decrease may indicate a transient activation state of memory B cells, that is, T-bet expression reflecting a temporary change in B cell status. Alternatively, considering that these frequencies were measured in PBMCs, it is possible that T-bet^hi^ B cells exhibit distinct homing patterns characterized by higher CXCR3 expression, leading to their migration into peripheral tissues, as reported in previous studies.^22^^,^^37^

Taken together, our results demonstrated that CD11c and FcRL5 double positivity can distinguish T-bet^hi^ B cells, both in *in silico* and *ex vivo*, which are robustly expanded by boost vaccinations.

### T-bet^hi^ B cells induced by multiple mRNA vaccinations are significantly affinity-matured and can rapidly differentiate into ASCs

**Figure 4:**
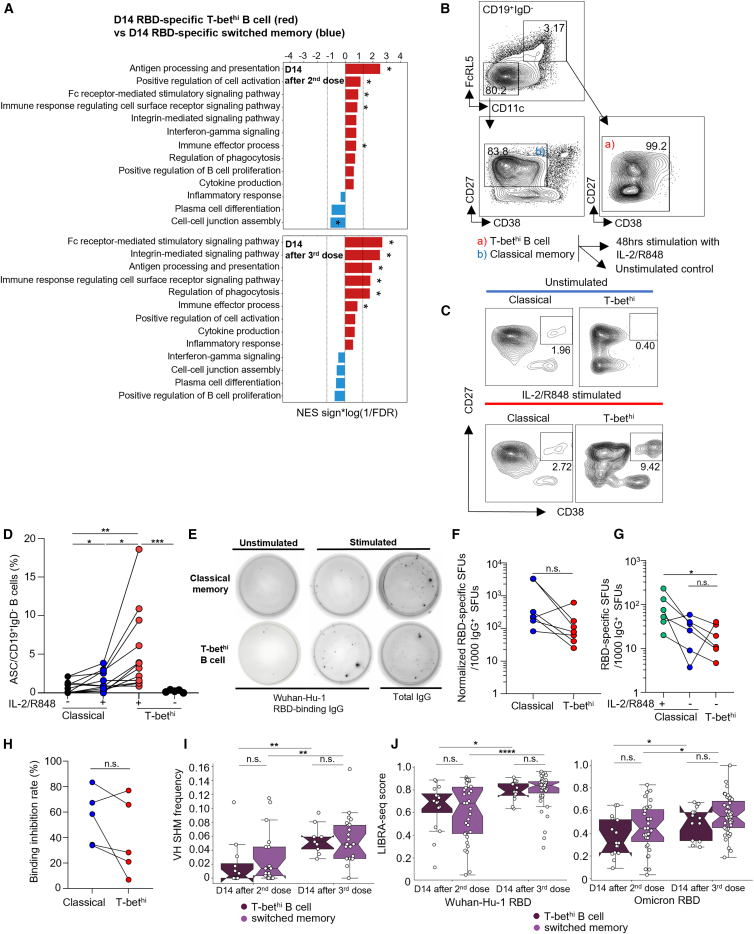
RBD-specific T-bet^hi^ B cells induced by multiple mRNA vaccinations are robustly affinity-matured and can rapidly differentiate into antibody-secreting cells (ASCs) (A) Gene set enrichment analysis (GSEA) showing that RBD-specific T-bet^hi^ B cells are significantly enriched in gene sets related to effector functions and activation, compared with RBD-specific switched memory B cells. Dotted lines indicate false discovery rate (FDR) of 0.05. (B) Gating strategy for fluorescence-activated cell sorting (FACS) of T-bet^hi^ and classical memory B cells. (C) Representative ASC frequencies among sorted T-bet^hi^ and classical memory B cells at 48 h after *in-vitro* polyclonal stimulation with IL-2 and R848. (D) Summary of ASC frequencies after stimulation of sorted T-bet^hi^ and classical memory B cells, showing more robust differentiation potentials of T-bet^hi^ B cells. (E) Representative data from IgG ELISPOT with sorted memory B cell subpopulations. (F) Summary of Wuhan-Hu-1 RBD-specific IgG SFUs per 1000 total IgG SFUs normalized by RBD-specific B cell frequency of each subset between classical and T-bet^hi^ B cells. (G) Summary of RBD-specific IgG SFUs per 1000 total IgG SFUs among stimulated classical memory, unstimulated classical memory and T-bet^hi^ B cells. (H) Surrogate RBD neutralization capacity of antibodies secreted by memory B cell subsets, in terms of binding inhibition rates. (I) Heavy chain somatic hypermutation frequency of RBD-specific memory B cells 14 days after the 2^nd^ or 3^rd^ vaccination, showing significant and comparable affinity maturation of T-bet^hi^ B cell. (J) LIBRA-seq scores calculated from RBD-tetramer ADT increases in both subpopulations following additional vaccine doses. Permutation test for GSEA terms in Figure 4A, Wilcoxon rank-sum test for Figures 4D–4H and Mann-Whitney U test for Figures 4I and 4J, ∗*p* < 0.05, ∗∗*p* < 0.01, ∗∗∗*p* < 0.001, ∗∗∗∗*p* < 0.0001; n.s., non-significant. Pooled data are represented as mean ± SD.

Since our data characterized T-bet^hi^ B cells as a distinct B cell subset that was robustly induced by multiple mRNA vaccinations, we next examined whether there were functional differences between T-bet^hi^ and switched memory B cells. In previous studies of T-bet^hi^ B cell in chronic diseases, it has appeared that T-bet^hi^ B cells exhibit reduced BCR signaling and capacity to differentiate into ASCs *in vitro.*^32^ However, recent studies show that in acute contexts and at early time-points, T-bet^hi^ B cells differentiate into alternative lineages—different from classical switched memory B cells—and participate in robust recall responses.^34^^,^^36^^,^^38^ Since we observed expansion of T-bet^hi^ B cells in response to multiple mRNA vaccinations, we evaluated differentially expressed genes (DEGs) between D14 RBD-specific T-bet^hi^ B cells and switched memory B cells. GSEA of the DEGs using gene ontology (GO) gene sets revealed significant enrichment of pathways related to antigen presentation, activation, proliferation, and effector functions in RBD-specific T-bet^hi^ B cells compared with RBD-specific switched memory B cells, suggesting that these cells may exhibit features of “effector memory” or reflect a recently activated B cell state (Figure 4A).

Based on these results, we next asked whether T-bet^hi^ B cells function as “effector memory” cells that can rapidly and effectively respond to cognate antigen challenge. A key effector function of memory B cells is their rapid differentiation into ASCs, which produce antigen-specific antibodies and remain quiescent in the absence of stimulation. To evaluate this function as a subset-specific property, we sorted equal numbers of isotype-switched T-bet^hi^ and classical switched memory B cells directly from D14 PBMCs obtained after the 2^nd^ or 3^rd^ vaccination. Each subset was then polyclonally stimulated with recombinant human IL-2 (10 ng/mL; Mabtech, Nacka Strand, Sweden) and R848 (1 μg/mL; Mabtech, Nacka Strand, Sweden) in combination with autologous T cells for 48 h. We sorted the T-bet^hi^ B cells subset using the gating strategy of CD11c and FcRL5 double positivity, based on our flow cytometry and LIBRA-seq findings (Figure 4B). First, we checked the ASC frequency in each stimulated memory subset (Figure 4C), and found that T-bet^hi^ B cells differentiated to CD27^+^CD38hi ASCs at a significantly higher frequency, compared to CD27^+^CD38^lo^ classical memory B cells (Figure 4D). This was in line with a previous study in human tonsil-derived FcRL5^+^ B cells.^21^ Furthermore, we asked whether this recall response could be observed in T-bet^hi^ B cells at later time points, and whether CD27 expression, a hallmark of classical memory B cell marker, affects recall responsiveness. To address this, we sorted CD27^+^ and CD27^−^ B cells within the CD11c^+^FcRL5^+^ B cell population from PBMCs of individuals 9 months after the 3^rd^ vaccination and stimulated them with IL-2/R848 for 48 h. Flow cytometric analysis after stimulation showed that both subpopulations underwent ASC differentiation with comparable frequencies (Figures S5D–S5G), demonstrating memory-like features of T-bet^hi^ B cells defined by CD11c and FcRL5, as well as the potential heterogeneity of this subset.

Since IL-2/R848 is a polyclonal B cell stimulus rather than RBD-specific, we performed IgG ELISPOT assays with Wuhan-Hu-1 SARS-CoV-2 RBD to assess whether differentiated ASCs included RBD-specific cells. Using PBMCs collected at D14 after the 2nd or 3rd vaccination, we applied the same sorting and stimulation protocols and observed significantly higher frequencies of RBD^+^ cells among T-bet^hi^ B cells compared with classical memory B cells (Figure S5H). To account for individual variation, RBD-specific SFUs were normalized to each donor’s RBD^+^ frequency. The total IgG^+^ SFUs per million cells were higher in T-bet^hi^ B cells (Figure S5I), yet normalized RBD-specific SFUs per 1000 IgG^+^ SFUs were similar between the subsets (Figures 4E and 4F), confirming that polyclonal stimulation yielded RBD-specific IgG-producing cells in both subsets. In addition, unstimulated T-bet^hi^ B cells from D14 PBMCs showed lower frequencies of RBD-specific ASCs than stimulated classical memory B cells and were comparable to unstimulated classical memory B cells (Figure 4G). This pattern suggests that T-bet^hi^ B cells more closely resemble stable memory B cells than recently activated cells readily or actively differentiate into plasmablasts.

Having established that polyclonal stimulation includes RBD-specific cells, we next performed surrogate neutralization assays on supernatants from polyclonally stimulated PBMCs of five triple-vaccinated individuals with breakthrough infection. We performed the same stimulation for 11 days, since it has been reported that this yields the highest immunoglobulin concentration.^39^ Our results showed that antibodies secreted by T-bet^hi^ B cells may have neutralization capacity comparable to antibodies secreted by classical memory B cells (Figure 4H), suggesting that T-bet^hi^ B cells may contribute to humoral response to cognate antigen challenges.

Since T-bet^hi^ B cells showed robust ability to differentiate into antigen-specific ASCs, we further examined their BCR properties. Somatic hypermutation (SHM) is the key mechanism that drives B cell affinity maturation against cognate antigens, and increased heavy-chain variable domain SHM (VH SHM) has been used as a hallmark of GC-driven memory B cell maturation.^40^ Interestingly, by the third vaccination, we found significantly increased VH SHM in RBD-specific T-bet^hi^ B cells, to a level comparable to that in switched memory B cells (Figure 4I). In accordance with this increased VH SHM, RBD-tetramer ADT was used to calculate a LIBRA-seq score, which is reportedly correlated with neutralization titer.^25^ The LIBRA-seq score showed significant increases for both Wuhan-Hu-1 RBD and Omicron (BA.1) RBD, in both D14 RBD-specific T-bet^hi^ B cells and switched memory B cells (Figure 4J).

Collectively, these results suggest that T-bet^hi^ B cells may play the role of effector memory B cells in early recall responses, i.e., rapidly and effectively respond to homologous challenges.

## Discussion

In the present study, we identified a transcriptionally distinct cluster exhibiting gene expression and surface receptor features of T-bet^hi^ B cells, among PBMCs from BNT162b2-vaccinated healthy individuals. GRN analysis revealed that T-bet^hi^ B cells were characterized by a distinct set of networks, with the TBX21 GRN being the most subset-specific regulatory network. We also found that T-bet^hi^ B cells comprise multiple clusters, including previously reported activated naive B cells,^17^ with transcriptional, phenotypic, and metabolic differences, suggesting potentially complex dynamics under the term “T-bet^hi^ B cells”. Additionally, we demonstrated that transcriptionally defined T-bet^hi^ B cells were delineated by a combination of CD11c^+^ and FcRL5^+^ *ex vivo*. Following the 2^nd^ and 3^rd^ BNT162b2 vaccinations, these B cells were robustly expanded among RBD-specific B cells, and mostly isotype-switched to IgG1. However, these B cells showed a sharp decline among RBD^+^ B cells after antigenic stimulation, which may indicate that they are transiently T-bet-upregulated activated B cells. Yet, because the frequencies were measured in peripheral blood, an alternative interpretation may also be that these findings align with previous reports showing that T-bet^hi^ B cells can be generated upon cognate antigen stimulation and subsequently compartmentalized in peripheral tissues such as the spleen, lymph nodes, and the lungs.^22^^,^^33^^,^^37^ Moreover, a recent human vaccine study reported that clonotypes of T-bet^hi^ B cells expanded by prior vaccination were represented in the repeated vaccinations and showed a positive correlation with long-term antibody response.^21^ Together, these findings may explain the observed decline as a result of migration, potentially contributing to long-term immunity in peripheral tissues where pathogens are frequently encountered.

In addition to gene regulatory networks, DEG analysis revealed that RBD-specific T-bet^hi^ B cells were enriched with gene sets related to effector functions. We further demonstrated that isotype-switched CD11c^+^FcRL5^+^ B cells more rapidly differentiated into CD27^+^CD38hi ASCs upon *in vitro* polyclonal stimulation, compared to isotype-switched CD27^+^CD38^lo^ classical memory B cells. Additionally, the 2^nd^ and 3^rd^ vaccinations significantly increased SHM in the heavy-chain V region of BCR and RBD-tetramer ADT among RBD-specific T-bet^hi^ B cells, to a comparable degree with switched memory B cells, suggesting robust T cell-dependent responses, in accordance with previous studies.^33^^,^^41^ Taken together, our results contribute to recently accumulating evidence that human T-bet^hi^ B cells may have effector memory features and involved in recall responses.

Conflicting results and various features reported by various studies of T-bet^hi^ B cells obscure a refined definition of this memory subset. In chronic infectious diseases, such as hepatitis C, AIDS, and malaria, T-bet^hi^ B cells show expanded frequency, but poor response to BCR stimulation and reduced capacity to produce cytokines and antibodies, which are features similar to antigen-specific T cell exhaustion.^42^ On the other hand, in autoimmune diseases, such as SLE, T-bet^hi^ B cells actively differentiate into autoantibody-secreting ASCs and are hyper-responsive to TLR.^17^ All described T-bet^hi^ B cells express T-bet, and share some surface phenotypes, such as CD11c. One hypothesis was that T-bet^hi^ B cells intrinsically show a poor response to BCR and hypersensitivity to non-antigen-specific cytokines and TLR, rendering them immunopathologic bystanders of the inflammation process. However, this hypothesis has been challenged by multiple mouse and human studies of T-bet^hi^ B cells in early recall responses, revealing robust antigen-specific proliferation and differentiation upon homologous challenge.^5^^,^^34^^,^^38^^,^^43^ Another hypothesis is that T-bet^hi^ B cells are effector memory B cells that rapidly respond to cognate antigen challenges, and that are exhausted by repetitive antigen stimulation and persisting inflammatory environments. This hypothesis is supported by recent data, including our present findings. However, as the evidence for its longevity as a long-lived memory subset remains incomplete, further investigations are needed to clarify whether T-bet^hi^ B cells constitutively express T-bet after the complete clearance of cognate antigens, to define their migration patterns to specific peripheral tissues, and to determine the duration of antigenic stimulation and inflammatory signals required to maintain this status. An alternative, but not mutually exclusive hypothesis, is that T-bet^hi^ B cells comprise multiple heterogeneous subsets, which may each prevail in different contexts. This is supported by our transcriptome data, and by the study of Reyes et al.*,*^43^ suggesting the need for further characterization of potential subsets and studies of the epigenetic, transcriptional, and functional changes of T-bet^hi^ B cells in various clinical settings.

The present study has several limitations. We showed that CD11c^+^FcRL5^+^ delineates T-bet^hi^ B cells; however, we cannot exclude the possibility that CD11c^+^FcRL5^–^ or CD11c^–^FcRL5^+^ B cells may at least partly share features of T-bet^hi^ B cells. Additionally, our BCR sequencing data were based on single-cell sequencing of multiplexed samples; thus, the depth of sequencing data hindered our ability to analyze clonal tracking along the longitudinal timeline. In line with this, we were unable to demonstrate that T-bet^hi^ B cells induced by repeated mRNA vaccinations are clonally related to newly generated ASCs or to long-lived memory B cells. Although we demonstrated rapid differentiation to ASCs and Wuhan-Hu-1-specific IgG production from T-bet^hi^ B cells, we did not link these findings to clinical parameters, such as correlates of protection in long-term humoral immunity and protective responses to variants. Further studies are warranted to explore the clinical relevance of this subset.

Overall, our present study elucidates the complex heterogeneity of human B cells with transcriptional and functional characterization of T-bet^hi^ B cells, from their priming to multiple recall responses following novel repetitive antigenic challenges in humans. These cells exhibit robust effector memory features, making them a potentially important immunological parameter for future vaccine design.

### Limitations of the study

We showed that CD11c^+^FcRL5^+^ delineates T-bet^hi^ B cells; however, we cannot exclude the possibility that CD11c^+^FcRL5^–^ or CD11c^–^FcRL5^+^ B cells may at least partly share features of T-bet^hi^ B cells. Additionally, our BCR sequencing data were based on single-cell sequencing of multiplexed samples; thus, the depth of sequencing data hindered our ability to analyze clonal tracking along the longitudinal timeline. In line with this, the number of RBD-specific B cells captured in our study was relatively low, resulting in fewer cells per subset and thereby weakening the statistical robustness of our interpretations. Although we demonstrated rapid differentiation to ASCs and Wuhan-Hu-1-specific IgG production from T-bet^hi^ B cell, we did not link these findings to clinical parameters, such as correlates of protection in long-term humoral immunity and protective responses to variants. This raises the alternative interpretation that T-bet expression may represent a transient change in certain subsets of activated memory B cells. In addition, we used a surrogate virus neutralization assay to evaluate antibodies secreted by B cells, which is not considered the gold standard for such assessments. Further studies are warranted to clarify the clinical relevance of this subset.

## Resource availability

### Lead contact

Further information and requests for resources and reagents should be directed to, and will be fulfilled by the lead contact, Su-Hyung Park (park3@kaist.ac.kr).

### Materials availability

Reagents generated in this study include streptavidin tetramers conjugated with biotinylated SARS-CoV-2 RBD (Acrobiosystems). Protocols to generate the tetramers are described in the method details. Recombinant antibodies were generated by Bionics Korea (Seoul, Korea) with curated light and heavy chain sequences from BCR-seq data (Table S3), available on request.

### Data and code availability

• LIBRA-seq data have been deposited at Gene Expression Omnibus (GEO, accession number: GSE315668) and are publicly available as of the date of publication. Accession numbers are listed in the key resources table.
• Code: This article does not report original code.
• Additional information: Any additional information required to reanalyze the data reported in this article is available from the lead contact upon request.

## Acknowledgments

This study was supported by grants from the 10.13039/501100003725National Research Foundation of Korea funded by the Korea government (MSIT) (RS-2023-00222762 to S.-H.P. and RS-2023-00219002 to S.-H.K.). This study was also supported by a grant from the Korea Health Technology R&D Project through the Korea Health Industry Development Institute (KHIDI), which is funded by the National Institute of Infectious Diseases, National Institute of Health, Republic of Korea (grant no. HD22C2045 to S.-H.P. and S.-H.K.), and was also supported by a Medical Scientist Training Program from the Ministry of Science & ICT of Korea (to J.L.) and a grant from Global Physician-Scientist Supporting Program by 10.13039/501100003710Korea Health Industry Development Institute (RS-2024-00410039, to J.L.).

## Author contributions

J.L., S.-H.K., S.-H. P., and E.-C.S. designed the study. J.L., Y.C., S.-D.C., J.-S.K., S.B., and J.S. collected clinical samples and information. J.L., S.S., Y.C., B.C., and I.J. carried out the experiments and collected the data. J.L., J.E.O., E.-C.S., S.-H.K., and S.-H.P. carried out the data analysis and interpretation. J.L., S.S., S.-H.K., and S.-H.P. wrote the manuscript with comments from all authors.

## Declaration of interests

The authors declare no competing interests.

## STAR★Methods

### Key resources table

**Table:** 

REAGENT or RESOURCE	SOURCE	IDENTIFIER
**Antibodies**		

BD Horizon™ BV421 Mouse Anti-Human CD27	BD biosciences	Cat# 562513; RRID: AB_11153497
BD Pharmingen™ Alexa Fluor® 700 Mouse Anti-Human CD38	BD biosciences	Cat# 560676; RRID: AB_1727472
APC Mouse Anti-Human CD19	BD biosciences	Cat# 555415; RRID: AB_398597
APC-H7 Mouse Anti-Human CD3	BD biosciences	Cat# 560176; RRID: AB_1645475
APC-H7 Mouse anti-Human CD14	BD biosciences	Cat# 560180; RRID: AB_1645464
Hu IgM BV510 G20-127 50Tst	BD biosciences	Cat# 563113; RRID: AB_2738010
Hu IgG BV786 G18-145 50Tst	BD biosciences	Cat# 564230; RRID: AB_2738684
BV605 Mouse Anti-Human IgD	BD biosciences	Cat# 563313; RRID: AB_2738134
PE-CF594 Mouse Anti-Human CD3	BD biosciences	Cat# 562280; RRID: AB_11153674
PE-CF594 Mouse Anti-Human CD56	BD biosciences	Cat# 564849; RRID: AB_2738983
BD Pharmingen™ Alexa Fluor® 700 Mouse Anti-Human CD38	BD biosciences	Cat# 560676; RRID: AB_1727472
BD Horizon™ BUV395 Mouse Anti-T-bet	BD biosciences	Cat# 568109; RRID: N/A
BD Horizon™ BUV805 Mouse Anti-Human CD19	BD biosciences	Cat# 568331; RRID: N/A
BD Horizon™ BUV737 Mouse Anti-Human CD27	BD biosciences	Cat# 612829; RRID: AB_2870151
PE anti-human CD307e (FcRL5) Antibody	Biolegend	Cat# 340304; RRID: AB_2104588
PE/Cyanine7 anti-human IgD Antibody	Biolegend	Cat# 348210; RRID: AB_10680462
APC/Cyanine7 anti-human CD56 (NCAM) Antibody	Biolegend	Cat# 362512; RRID: AB_2564085
TotalSeq™-C0050 anti-human CD19 Antibody	Biolegend	Cat# 302265; RRID: AB_2800741
TotalSeq™-C0154 anti-human CD27 Antibody	Biolegend	Cat# 302853; RRID: AB_2800747
TotalSeq™-C0181 anti-human CD21 Antibody	Biolegend	Cat# 354923; RRID: AB_2800953
TotalSeq™-C0394 anti-human CD71 Antibody	Biolegend	Cat# 334125; RRID: AB_2800885
TotalSeq™-C0053 anti-human CD11c Antibody	Biolegend	Cat# 371521; RRID: AB_2801018
TotalSeq™-C0140 anti-human CD183 (CXCR3) Antibody	Biolegend	Cat# 353747; RRID: AB_2800949
TotalSeq™-C0144 anti-human CD185 (CXCR5) Antibody	Biolegend	Cat# 356939; RRID: AB_2800968
TotalSeq™-C0829 anti-human CD307e (FcRL5) Antibody	Biolegend	Cat# 340309; RRID: AB_2819969
TotalSeq™-C0389 anti-human CD38 Antibody	Biolegend	Cat# 303543; RRID: AB_2800758
TotalSeq™-C0100 anti-human CD20 Antibody	Biolegend	Cat# 302363; RRID: AB_2800743
TotalSeq™-C0831 anti-human CD138 (Syndecan-1) Antibody	Biolegend	Cat# 352327; RRID: AB_2814282
TotalSeq™-C0136 anti-human IgM Antibody	Biolegend	Cat# 314547; RRID: AB_2800835
TotalSeq™-C0375 anti-human IgG Fc Antibody	Biolegend	Cat# 410727; RRID: AB_2801087
TotalSeq™-C0384 anti-human IgD Antibody	Biolegend	Cat# 348245; RRID: AB_2810553
TotalSeq™-C0047 anti-human CD56 (NCAM) Antibody	Biolegend	Cat# 362559; RRID: AB_2801002
TotalSeq™-C0049 anti-human CD3 Antibody	Biolegend	Cat# 344849; RRID: AB_2814272
TotalSeq™-C0081 anti-human CD14 Antibody	Biolegend	Cat# 301859; RRID: AB_2800736
TotalSeq™-C0896 anti-human CD85j (ILT2) Antibody	Biolegend	Cat# 333725; RRID: AB_2814226
PerCP/Cyanine5.5 anti-human CD21 Antibody	Biolegend	Cat# 354908; RRID: AB_2561544
APC anti-human CD307e (FcRL5) Antibody	Biolegend	Cat# 340306; RRID: AB_2564327
CD11c Antibody, FITC	Invitrogen	Cat# 11-0116-42; RRID: AB_10718106
CD14 Monoclonal Antibody (61D3), PE-eFluor™ 610, eBioscience™	Invitrogen	Cat# 61-0149-42; RRID: AB_2574534

**Biological samples**		

Human peripheral blood mononuclear cells	Asan Medical Center	N/A

**Chemicals, peptides, and recombinant proteins**		

TotalSeq™-C0952 PE Streptavidin	Biolegend	Cat# 405263
TotalSeq™-C0953 PE Streptavidin	Biolegend	Cat# 405265
Biotinylated SARS-CoV-2 Spike RBD, His,Avitag™ (MALS verified)	Acrobiosystems	Cat# SPD-C82E9
Biotinylated SARS-CoV-2 Spike RBD, His,Avitag™ (B.1.1.529/Omicron) (MALS verified)	Acrobiosystems	Cat# SPD-C82E4-200

**Critical commercial assays**		

Chromium Next GEM Single Cell 5′ Kit v2, 16 rxns	10X Genomics	Cat# PN-1000263
Chromium Next GEM Chip K Single Cell Kit, 16 rxns	10X Genomics	Cat# PN-1000287
Dual Index Kit TN Set A, 96 rxns	10X Genomics	Cat# PN-1000250
Dual Index Kit TT Set A, 96 rxns	10X Genomics	Cat# PN-1000215
Chromium Single Cell Human BCR Amplification Kit, 16 rxns	10X Genomics	Cat# PN-1000253
ELISpot Flex: Human IgG (ALP)	MABTECH	Cat# 3850-2A
SARS-CoV-2 Surrogate Virus Neutralization Test (sVNT) Kit	GenScript	Cat# L00847-A
SARS-CoV-2 Spike S1-RBD IgG&IgM ELISA Detection Kit	GenScript	Cat# L00845

**Deposited data**		

LIBRA-seq (RNA, feature barcode and BCR)	This paper	Accession# GSE315668

**Software and algorithms**		

Souporcell v. 2.5	Heaton et al.^44^	N/A
Scanpy v. 1.9.2	Wolf et al.^45^	RRID: SCR_018139
SoupX v. 1.6.0	Young et al.^46^	RRID: SCR_019193
Scrublet v. 0.2.3	Wolock et al.^47^	RRID: SCR_018098
Harmonypy v. 0.0.9	Korsunsky et al.^48^	RRID: SCR_022798
pyWNN	Hao et al.^29^	https://github.com/dylkot/pyWNN
ChangeO v. 1.3.0	Gupta et al.^49^	RRID: SCR_023986
SHazaM v. 1.1.1	Gupta et al.^49^	RRID: SCR_024301
Gseapy v. 1.0.6	Fang et al.^50^	https://gseapy.readthedocs.io/en/latest/
SCOPer v. 1.3.0	Nouri et al.^51^	https://scoper.readthedocs.io/en/stable/
FlowJo software v. 10.8	Treestar	N/A
GraphPad Prism v. 8.0	GraphPad Software	N/A
SoftMax Pro software	Molecular Devices	RRID: SCR_012780

**Other**		

Lymphocyte separation medium	Corning	Cat# 25-072-CV
Dimethyl sulfoxide	Sigma-Aldrich	Cat# D8418
Fetal bovine serum	Corning	Cat# 35-010-CV
Penicillin-Streptomycin Solutions	WELGENE	Cat# LS202-02
LIVE/DEAD red fluorescent reactive dye	Invitrogen	Cat# L34972
LIVE/DEAD near-IR fluorescent reactive dye	Invitrogen	Cat# L34976
FoxP3 staining buffer kit	Invitrogen	Cat# 00-5523-00
LSR II flow cytometer	BD Biosciences	N/A
FACSAria™ II cell sorter	BD Biosciences	N/A
Deoxyribonuclease I, from bovine pancreas	Sigma-Aldrich	Cat# D5025
CD19 MicroBead Human	Miltenyi Biotec	Cat# 130-050-301
FcR Blocking Reagent, human	Miltenyi Biotec	Cat# 130-059-901
AID iSpot Spectrum	Advanced Imaging Devices GmbH	ELR088IFL
BioTek Epoch Microplate Spectrophotometer	Biotek	Cat# 20081918
Pan T cell isolation kit, human	Miltenyi Biotec	Cat# 130-096-535

### Experimental model and study participant details

Medical personnel at Asan Medical Center (AMC) who self-identified healthy and tested negative for COVID-19 at the time of recruitment volunteered to participate in this study, and gave written informed consent. The mean age was 34. Forty-eight percent of these subjects were male and fifty-two percent of these subject were female. All of the subjects were Asian Korean. Peripheral blood was drawn using EDTA tubes, at multiple time-points along the vaccination schedule. All human samples were collected with approval from the Institutional Review Board of Asan Medical Center (IRB approval number: AMC S2020-3148-0001). Detailed characteristics of individuals used in assays of the present study can be found in Table S1.

### Method details

#### Lymphocyte isolation

Medical personnel volunteered to participate in this study, and gave written informed consent. Peripheral blood was drawn using EDTA tubes, at multiple time-points along the vaccination schedule, at Asan Medical Center. Mononuclear cells were separated from the blood using lymphocyte separation medium (Corning) and were either used for experiments, or stored for later use by cryopreservation in a liquid nitrogen tank, in freezing medium comprising 70% fetal bovine serum (FBS; Corning), 20% RPMI, and 10% dimethyl sulfoxide (Sigma-Aldrich). All human samples were collected with approval from the Institutional Review Board of Asan Medical Center (IRB approval number: AMC S2020-3148-0001). Characteristics of individuals used in assays of the present study can be found in Table S1.

#### SARS-CoV-2 RBD-specific tetramer

For antigen-tetramer generation, fluorochrome-conjugated streptavidin (Invitrogen) was incrementally added to biotinylated SARS-CoV-2 RBD (Wuhan-Hu-1 and BA.1) in PBS (200 μg/mL; Acrobiosystems). After each addition step, the mixture was incubated for 20 minutes at 4°C with gentle mixing. The final molar ratio of RBD to streptavidin was 4:1. Decoy streptavidin (Invitrogen) was also incrementally added to biotin in PBS (0.1 mg/ml; Sigma-Aldrich) until reaching a final 4:1 molar ratio of biotin to streptavidin. For LIBRA-seq, oligonucleotide-conjugated fluorochrome-conjugated streptavidin (Biolegend) was used for antigen-tetramer generation, and subsequently used for cell staining.

#### Indirect ELISA for recombinant antibodies

SARS-CoV-2 Spike S1-RBD IgG & IgM ELISA Detection Kit (GenScript, L00845) was used to quantify recombinant antibodies binding to SARS-CoV-2 RBD proteins. 100μl of positive control, negative control, and serially diluted antibodies (initial concentration at 104 mg/ml, followed by 6 additional five-fold serial dilutions) were added to each well of pre-coated plate with SARS-CoV-2 Spike S1-RBD antigen and incubated for 30 minutes at 37°C. Plates were then washed 4 times with wash buffer and subsequently incubated with 100μl of HRP conjugated Mouse anti-Human IgG for 15 minutes at 37°C. Following this, plates were then washed 4 times with wash buffer and incubated with 100 μl of TMB 3,3′,5,5′-tetramethylbenzidine (TMB) substrate for 15 minutes at room temperature. The reaction was stopped with 50μl of Stop Solution, and absorbance was measured at 450 nm using an ELISA microplate reader (SpectraMax Mini, Molecular Devices) with SoftMax Pro software (RRID: SCR_012780) for analysis.

#### Flow cytometry and immunophenotyping

Cryopreserved cells were thawed, and then a single-cell suspension was prepared in RPMI with 10% FBS for staining. First, CD19^+^ B cells were positively sorted using CD19 microbeads (Miltenyi Biotec) through a MACS MS column (Miltenyi Biotec). Next, these cells were stained with Fc receptor blocker (Miltenyi Biotec) for 10 minutes at 4°C. The cells were subsequently stained with decoy tetramer (5 μl, 60 nM)—i.e., PE-Cy7-conjugated streptavidin molecules (Thermo Fischer Scientific) bound with biotin (Sigma-Aldrich)—to block non-specific B cells, or B cells that bind streptavidin, phycoerythrin (PE), or biotin, for 10 minutes at room temperature. In the next step, the cells were stained with PE-conjugated streptavidin (5 μl, 60 nM) bound with SARS-CoV-2 RBD, for 30 minutes at 4°C.

After washing, the cells were stained with fluorochrome-conjugated antibodies against surface markers, for 20 minutes at room temperature. Next, the cells were either sorted for subsequent sequencing using a FACSAria II cell sorter (BD Biosciences), or analyzed by flow cytometry using an LSR II instrument and FACSDiva software (BD Biosciences). Data were analyzed using FlowJo software (Treestar).

#### CITE-seq with SARS-CoV-2 RBD-specific tetramer (LIBRA-seq) and analysis

The stained cells were sorted into tetramer^+^ cells and tetramer^−^ cells and cells from 4 individuals were pooled to be included in a single gem. Tetramer^−^ cells were titrated up to a total of 100,000 cells, and then mixed with tetramer^+^ cells. This cell mixture was resuspended in 2% bovine serum albumin (BSA; Bovogen) in PBS for CITE-seq antibody staining. The cells were incubated with Fc receptor blocker (Miltenyi Biotec) at 4°C, and then stained with prepared CITE-seq antibody mixture and incubated at 4°C for 30 minutes. Next, the cells were washed 3 times, and resuspended to 1000 cells/μl, in a volume of 40–100 μl for subsequent library preparation. Transcriptome, antibody-derived tag, and BCR libraries were separately generated using 10X Genomics kits. All samples were sequenced using an Illumina HiSeq 2500, with an average of over 20 million 100-bp paired-end reads. FASTQ files were mapped to the human genomic DNA reference (GRCh38) using cellranger-6.1.2. After alignment, individual annotation was performed using Souporcell v2.5,^44^ and downstream computational analysis was conducted with Python and R packages. Raw read count matrices were first processed using SoupX v1.6.0^46^ to quantify and remove the effects of ambient RNA contamination. Next, the raw count data were processed into anndata objects using Scanpy v1.9.2^45^ with the standard workflow, and doublets were removed using Scrublet v0.2.3.^47^ In quality control, cells were filtered out if they contained >7000 or <400 genes, and the mitochondrial content cut-off was set at <10%. After normalization and log-transformation, highly variable genes were selected with a minimal mean of 0.0125, maximum mean of 3.5, and minimal dispersion of 0.5. Batch correction was performed using Harmony v.0.0.9,^48^ and 15 transformed principal components were used for clustering UMAP construction. Clustering was performed with the Leiden algorithm at a resolution of 0.34, and each cluster was annotated according to its mean expression levels of representative genes. Gene regulatory networks were derived by SCENIC^26^ using all B cells acquired after quality control process. Details of all derived GRNs can be found in Table S2. Subclustering analysis of the T-bet^hi^ B-cell cluster was performed using weighted-nearest neighbor (WNN) in pyWNN.^29^ GSEA was performed for each comparison group using Gseapy v.1.0.6^50^ and detailed information can be found in Table S3. BCR-filtered sequence data were processed with ChangeO v.1.3.0^49^ for VDJ gene annotation, and clone assignment was performed using SCOPer v.1.3.0.^51^ Mutation analysis of processed BCR sequences was performed using SHazaM v.1.1.1.^49^

#### *In vitro* stimulation of memory B cells

For polyclonal stimulation, memory B cells were sorted into T-bet^hi^ B cell and classical memory B cells using the following antibodies: anti-CD27-BV421 (clone M-T271; BD Biosciences), anti-CD11c-FITC (clone 3.9; Invitrogen), anti-CD38-PerCP-Cy5.5 (clone HIT2; BD Biosciences), anti-FcRL5-PE (clone 509f6; Biolegend), anti-IgD-PE-Cy7 (clone IA6-2; Biolegend), anti-CD19-APC (clone HIB19; BD Biosciences), anti-CD3-APC-H7 (clone SK7; BD Biosciences), anti-CD14-APC-H7 (clone MφP9; BD Biosciences) and anti-CD56-APC-Cy7 (clone 5.1H11; Biolegend). The sorted cells were then incubated with autologous T cells, at a density of 1 × 10^6^ cells/ml in culture plates (SPL Life Sciences) for 48 hours, with IL-2 (10 ng/ml; MABTECH) and R848 (1 μg/ml; MABTECH), as previously described. After incubation, the cells were harvested and stained using the same panel of antibodies used for sorting, to calculate the frequencies of antibody-secreting cell-differentiated memory B cells. Normalization was done by dividing the RBD-specific SFUs/1000 total IgG SFUs by corresponding frequency of RBD^+^ in each subset.

#### IgG ELISPOT using *in vitro* stimulated memory B cells

Memory B cells were sorted and incubated as described above, and then the cells were harvested and vigorously washed to remove secreted antibody. Next, the cells were transferred into type MSIP PVDF plates (Millipore) that had been pre-coated with anti-human IgG antibody. These plates were incubated for 24 hours for antibody secretion. Subsequently, the cells were removed from the plates. To the wells, we added biotinylated RBD (Wuhan-Hu-1, Acrobiosystems, 1 μg/ml) or anti-IgG monoclonal antibody (clone MT78/145, MABTECH) as a positive control, followed by a 2-hour incubation at room temperature. Next, streptavidin-ALP was added to every well, followed by a 1-hour incubation at room temperature. Finally, BCIP/NBT (5-bromo-4-chloro-3-indolyl-phosphate/nitro blue tetrazolium, MABTECH) substrate was added, and then the plates were incubated for 5–10 minutes until spots emerged. After spots developed, the plates were dried overnight in the dark, and spots were counted using AID iSpot Spectrum (Advanced Imaging Devices GmbH).

#### Surrogate virus neutralization test

Memory B cells were sorted and cultured for 11 days.^39^ Next, the supernatant fluid was collected, serially diluted, and used as serum samples with the SARS-CoV-2 surrogate virus neutralization test kit (GenScript), following the manufacturer’s protocol. We measured the OD_450_ values using a BioTek Epoch Microplate Spectrophotometer (BioTek). The inhibition percentage was calculated following the manufacturer’s protocol.

$$\text{Inhibition}=\left(1-\frac{\text{OD}\;\text{value}\;\text{of}\;\text{sample}}{\text{OD}\;\text{value}\;\text{of}\;\text{negative}\;\text{control}}\right)\times 100\;\left(\%\right)$$

The inhibition percentage cut-off was set as 30%, according to the protocol.

#### Statistical analyses

Statistical analyses were performed using Python version 3.7.0 (Van Rossum, G., & Drake Jr, F. L., 1995). Continuous variables were compared using the Mann-Whitney U test (Wilcoxon signed-rank test for paired data). Kruskal-Wallis test was performed for trend analysis of data with more than 2 groups. Correlations between continuous variables were assessed using Pearson correlation analysis. Wilcoxon test was used for differentially expressed genes analysis and permutation test was done for GSEA. Significance was defined as a *P* value of <0.05 or a false discovery rate of <0.05. ∗*P* < 0.05, ∗∗*P* < 0.01, ∗∗∗*P* < 0.001, ∗∗∗∗*P* < 0.0001; n.s., non-significant.

## Supplemental information

Document S1. Figures S1–S5 and Table S1

Table S2. The full list of regulons derived from the LIBRA-seq dataset by SCENIC

Table S3. GSEA results shown in Figure 4A and BCR sequences used to make recombinant antibodies tested in Figure 1G
