# Supplementary material for: Robust effector memory features of human T-bethi B cells induced by repeated mRNA vaccination
Source: iScience. 2026 Jan 27;29(2):114804. doi: 10.1016/j.isci.2026.114804 (PMC12915279; doi:10.1016/j.isci.2026.114804)
Supplement: Document S1. Figures S1–S5 and Table S1 [file mmc1.pdf]

iScience, Volume *29*

Supplemental information

Robust effector memory features of human T-bet^{hi} B cells induced by repeated mRNA vaccination

Jeongsoo Lee, Seunghwan Son, Youseung Chung, Sung-Dong Cho, Ji-Soo Kwon, Seongman Bae, Joon Seok, Baekgyu Choi, Inkyung Jung, Ji Eun Oh, Eui-Cheol Shin, Sung-Han Kim, and Su-Hyung Park

Supplementary Fig. 1. Overview of LIBRA-seq analysis parameters

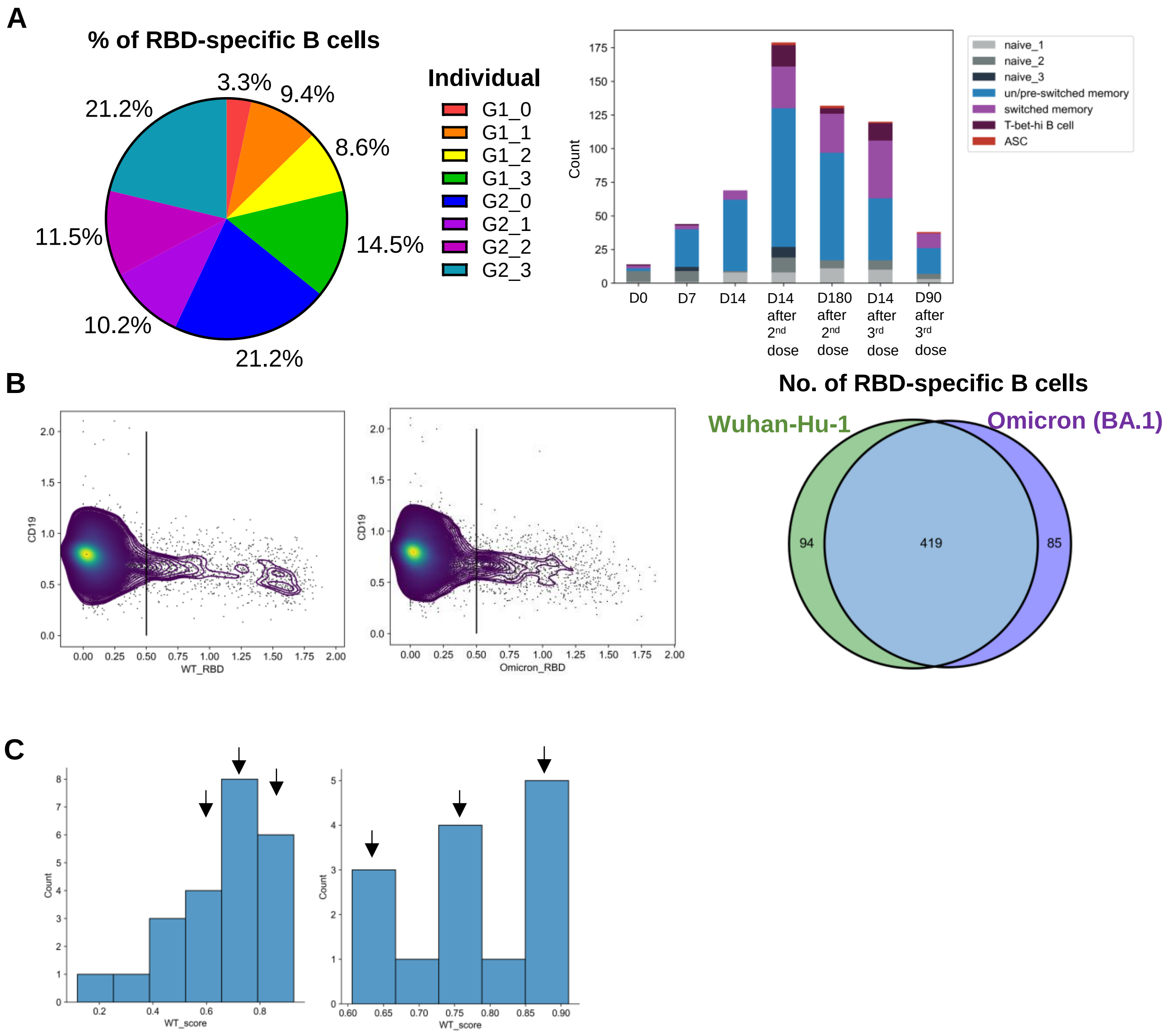

Supplementary Fig. 2. Gene regulatory network analysis and T-bet regulon genes

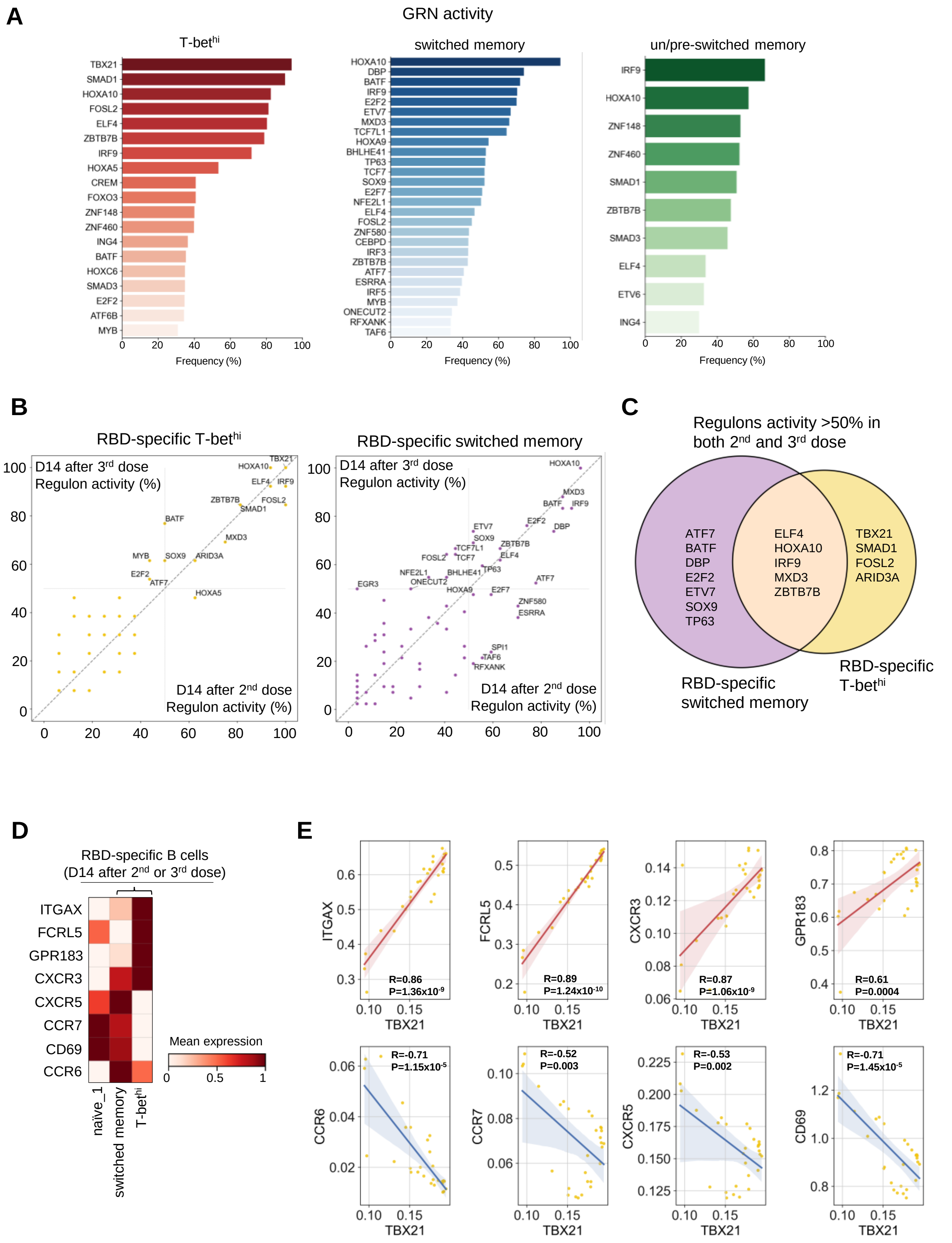

Supplementary Fig. 3. Characteristics of subclusters of T-bet^{hi} B cells

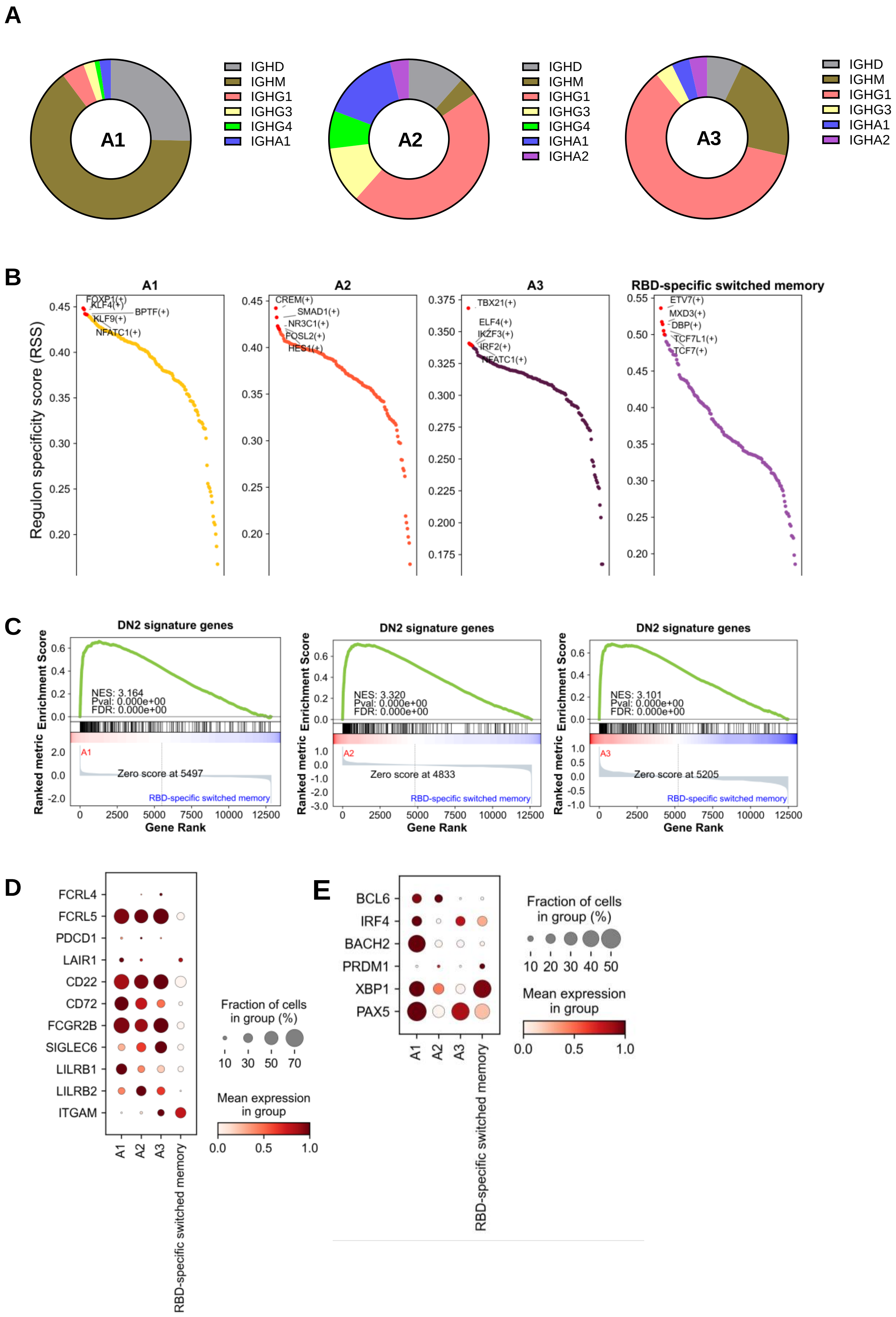

Supplementary Fig. 4. Machine learning analysis for antibody-derived tags of B cell clusters

A

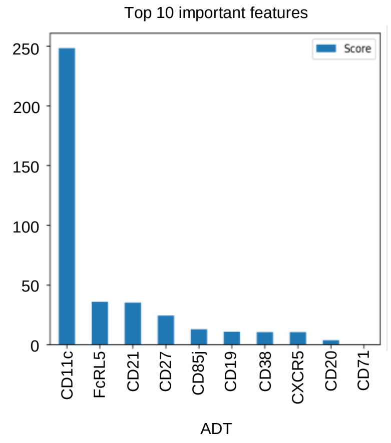

B

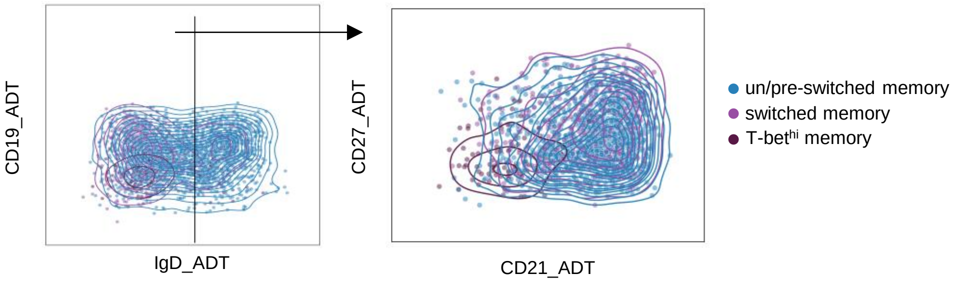

Supplementary Fig. 5. Phenotypic analysis of T-bet^{hi} B cells

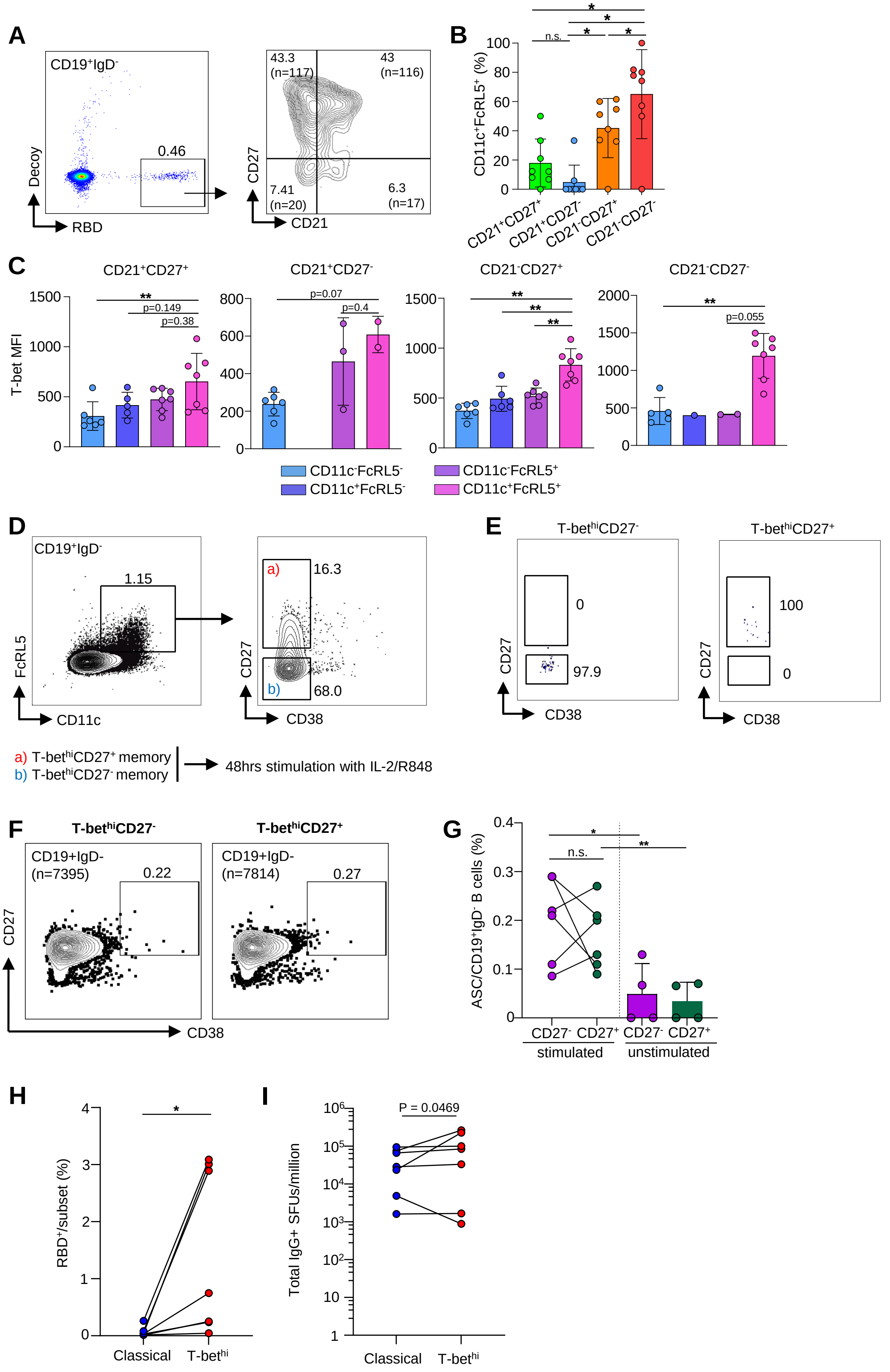

Supplementary Table 1. Deidentified individual information used for each analysis.

Code	Age	Sex	Vaccine	Used assay				
				Flow cytometry (kinetics/phenotypes)	Flow cytometry (activation culture)	LIBRA-seq	IgG ELISPOT	Supernatant antibody assay
K1	32	M	BNT162b2		V		V	V
K2	43	F	BNT162b2				v	V
K3	33	M	BNT162b2					V
K4	25	F	BNT162b2				V	
K5	55	F	BNT162b2					V
K6	44	M	BNT162b2		V		V	V
V5	43	M	BNT162b2	V				
V13	53	M	BNT162b2			V		
V15	29	F	BNT162b2	V				
V18	32	M	BNT162b2	V			V	
V19	34	M	BNT162b2	V				
V21	38	M	BNT162b2	V	V		V	
V22	48	M	BNT162b2		V			
V23	28	F	BNT162b2	V				
V24	29	M	BNT162b2	V				
V25	33	M	BNT162b2				V	
V26	38	F	BNT162b2				V	
V27	26	M	BNT162b2	V			V	
V28	32	F	BNT162b2	V				
V29	31	F	BNT162b2	V				
V32	53	M	BNT162b2		V			
V33	27	F	BNT162b2		V	V		
V34	35	M	BNT162b2	V	V	V	V	
V35	38	F	BNT162b2	V			V	
V37	42	M	BNT162b2	V		V	V	
V41	30	F	BNT162b2				V	
V42	32	F	BNT162b2	V				
V45	26	F	BNT162b2	V	V	V		
V46	39	M	BNT162b2	V			V	
V49	26	M	BNT162b2	V				
V51	35	M	BNT162b2	V			V	
V52	26	F	BNT162b2	V			V	
V54	32	F	BNT162b2				V	
V56	27	F	BNT162b2	V	V		V	
V58	36	F	BNT162b2	V	V		V	

Supplementary Figure 1. (A) Proportions of individuals sampled RBD-binding B originated from and tetramer specificity frequencies of RBD-binding B cells (left) and frequency of clusters among RBD-binding B cells according to the timepoint (right). **(B)** In-silico gating cutoff for RBD-binding B cells using tetramer ADTs (left) and Venn diagram summary of Wuhan-Hu-1 and BA.1-specific B cells (**C**) LIBRA-seq score distribution for Wuhan-Hu-1 RBD tetramer and selected bins for sequences used to make recombinant antibodies, black arrows indicate the bins for sequence selection to generate recombinant antibodies.

Supplementary Figure 2. (A) Gene regulatory networks (GRN) activity in total T-bet^{hi}, switched and un/pre-switched memory B cells. GRNs positive in over 30% of cells in the cluster are shown. **(B)** GRN activity of RBD-specific T-bet^{hi} B cell (left) and RBD-specific switched memory B cells (right) at day 14 after the 2nd and 3rd vaccination doses. **(C)** Venn diagram of regulons active in over 50% of each subpopulation. **(D)** Receptor-binding domain (RBD)-specific T-bet^{hi} B cell show higher expressions of ITGAX and FCRL5 and chemokine receptor genes, defining a distinct trafficking pattern. **(E)** TBX21 expression in RBD-specific T-bet^{hi} B cell is significantly correlated with ITGAX, FCRL5, and chemokine receptor genes.

Supplementary Figure 3. (A) Isotype frequencies of each subcluster of D14 T-bet^{hi} B cell. **(B)** Regulon specificity score showing specific GRNs in each subcluster of T-bet^{hi} B cell. **(C)** Double-negative 2 (DN2) signature genes are significantly enriched in all three subclusters, compared with RBD-specific switched memory B cells. **(D)** Inhibitory receptor expression in T-bet^{hi} B-cell subclusters and RBD-specific switched memory B cells. **(E)** Expressions of key transcription factors involved in B-cell differentiation.

Supplementary Figure 4. (**A)** Top 10 antibody-derived tag (ADT) features distinguishing the T-bet^{hi} B-cell cluster, selected by a machine learning model. **(B)** In-silico gating strategy to sort IgD-negative B cells.

Supplementary Figure 5. (A) Gating strategy of RBD^+ B cells among $CD19^+IgD^-$ cells and 4 subpopulations by CD21 and CD27 expression. **(B)** The frequency of $CD11c^+FcRL5^+$ B cells among 4 subpopulations. **(C)** T-bet MFI level in each subpopulation by CD21 and CD27. **(D)** Gating strategy for fluorescence-activated cell sorting (FACS) of T-bet^{hi} and sorting by CD27 expression. **(E)** Representative of data of purity check after sorting. **(F)** Representative plots of flow cytometry analysis of B cells after stimulation. **(G)** The frequency of ASCs among $CD19^+IgD^-$ B cells after 48-hour stimulation. **(H)** The frequency of RBD^+ cells among each subset from individuals whose B cells were used for IgG ELISPOT. **(I)** Total IgG SFUs (spot-forming unit) for IgG ELISPOT represented in Figure 4E-F. $*P < 0.05$, $**P < 0.01$, $***P < 0.001$, $****P < 0.0001$; n.s., non-significant. Pooled data are represented as mean ± SD.

Supplementary Table 1. Deidentified individual information used for each analysis.

Supplementary Table 2. The full list of regulons derived from the LIBRA-seq dataset by SCENIC.

Supplementary Table 3. GSEA results shown in Figure 4A and BCR sequences used to make recombinant antibodies tested in Figure 1G.
